# Contagion of Cholera

**Published:** 1849

**Authors:** 


### Article IX.—*Contagion of Cholera.*

The following instance is quoted as an example of the propagation of cholera by contagion. "The British frigate, 'Topaz,' touched at the Isle of France in 1829, and conveyed thither cholera, which spread rapidly throughout the island, prolonging its ravages for four months, sparing neither age, sex, nor rank, although the deaths were more numerous among the black population. No room was left for doubt that the disease had, in this instance propagated itself directly by contagion. Six thousand individuals perished during the first six weeks of its appearance. When this ship arrived, the Island was in in a healthy state, and had been free from any epidemic illness for an unusually long period." *Dub. Med. Press.* in *Med. News.*

